# Nonlinear Hyperparameter Optimization of a Neural Network in Image Processing for Micromachines

**DOI:** 10.3390/mi12121504

**Published:** 2021-11-30

**Authors:** Mingming Shen, Jing Yang, Shaobo Li, Ansi Zhang, Qiang Bai

**Affiliations:** 1 School of Mechanical Engineering, Guizhou University, Guiyang 550025, China; 15985146314@163.com (M.S.); jyang23@gzu.edu.cn (J.Y.); zhangas@gzu.edu.cn (A.Z.); 18798824036@163.com (Q.B.); 2 School of Mechanical & Electrical Engineering, Guizhou Normal University, Guiyang 550025, China; 3 State Key Laboratory of Public Big Data, Guizhou University, Guiyang 550025, China

**Keywords:** deep neural network, hyperparameters, multiparameter mathematical correlation model, image processing

## Abstract

Deep neural networks are widely used in the field of image processing for micromachines, such as in 3D shape detection in microelectronic high-speed dispensing and object detection in microrobots. It is already known that hyperparameters and their interactions impact neural network model performance. Taking advantage of the mathematical correlations between hyperparameters and the corresponding deep learning model to adjust hyperparameters intelligently is the key to obtaining an optimal solution from a deep neural network model. Leveraging these correlations is also significant for unlocking the “black box” of deep learning by revealing the mechanism of its mathematical principle. However, there is no complete system for studying the combination of mathematical derivation and experimental verification methods to quantify the impacts of hyperparameters on the performances of deep learning models. Therefore, in this paper, the authors analyzed the mathematical relationships among four hyperparameters: the learning rate, batch size, dropout rate, and convolution kernel size. A generalized multiparameter mathematical correlation model was also established, which showed that the interaction between these hyperparameters played an important role in the neural network’s performance. Different experiments were verified by running convolutional neural network algorithms to validate the proposal on the MNIST dataset. Notably, this research can help establish a universal multiparameter mathematical correlation model to guide the deep learning parameter adjustment process.

## 1. Introduction

Deep neural networks are widely used in the field of image processing for micromachines. For example, a deep neural network model can be used in detecting the 3D shapes of droplets online in the process of microelectronic high-speed dispensing or droplets formed by microfluidic chips [1]. The convolutional neural network (CNN) has been proven to have better performance in hyperspectral image (HSI) classification than traditional methods, while HSI classification of remote sensing makes full use of high-altitude detection equipment [2]. In particular, neural networks (DNN) have recently proven their effectiveness when applied to image recognition to extract the meaningful information from sensors in a smart tactile sensing system of micromachines [3]. The outstanding performance of each of the above deep neural networks is inseparable from the excellent design of the system architecture and the elaborate selection of hyperparameter values. Hyperparameters, such as the learning rate (*lr*), batch size (*m*), convolution kernel size (*ke*), and dropout rate (*q*), determine the performance of a deep neural network model, which has a significant impact on the deep-learning-based system of micromachines. There is an urgent need for a systematic, mathematical study of hyperparameters in deep neural networks and to explain how to set the hyperparameters to get the best performance of the model. 

However, choosing the right value for a given hyperparameter is very tricky, because doing so depends not only on the programmer’s experience level, but also on the ability of the model to learn from each round of value experiments. In general, the strategy of combining a grid search and a manual search is adopted for hyperparameter configuration [4,5,6]. The manual search method has difficulty reproducing the obtained experimental results, and the grid search strategy is only suitable for cases involving a small number of hyperparameters in practice, and is performed utilizing an expensive trial-and-error approach. Although random search [7] has been proposed, and researchers can identify good hyperparameter values with accuracy by random sampling from the search space, random search has the possibility of performing repeated searches with the same hyperparameters. The Bayesian-optimization-based algorithms improve the random-search-based optimization process, and can find better hyperparameters more quickly [8,9,10,11]. However, these methods can only perform static hyperparametric searches. Hyperparameter optimization algorithms are proposed based on reinforcement learning, such as the particle swarm optimization method [12,13], multiparameter optimization method [14,15], and dynamic optimization method [16,17,18]. These algorithms have been proven to be beneficial for optimizing structural parameters. However, hyperparameters such as *lr*, *q*, *m,* and *ke* still need to be manually set by the user. Determining the optimal values of these hyperparameters has always been a key objective in the field of deep learning research. At present, the configuration and optimization of hyperparameters mostly rely on experiments and experience, and researchers generally select hyperparameter values within certain ranges based on others’ experiences. This approach lacks a systematic description of the effects of multiple hyperparameters on the performances of deep learning models based on mathematical principles and convolution theory. 

Here, after the Introduction, the authors present a review of the literature investigating the relationship between hyperparameter tuning and model performance. These studies relate to the interpretability of deep CNNs, the optimizer of the deep neural network model and commonly used hyperparameters. The mathematical equations between individual hyperparameters (*lr*, *m*, *q,* and *ke*) and model performance are derived separately based on the previous work. Based on this, the authors attempt to integrate the four hyperparameters and derive mathematical relationships to account for the interactive effects of these hyperparameters on model performance. After completing the mathematical derivation, the authors first conduct single-hyperparameter cross-validation experiments on three models while considering the effects of individual hyperparameters. Then, they select the optimal hyperparameter combination based on the above-mentioned results and conduct multiparameter validation experiments using the three models again. Finally, the conclusions are drawn in the last part. Combining mathematical derivation and experimental validation, the authors want to give a systematic, mathematical study to hyperparameters in deep neural networks and establish a universal multiparameter mathematical correlation model to guide the hyperparameters adjustment process.

The main contributions of this paper are as follows:

1. The correlations among the *lr*, *m*, *q,* and *ke* are explained through a theoretical derivation. A generalized mathematical model is established to guide the parameter adjustment process for the examined deep learning model.

2. The effective influences of *lr*, *m*, *q*, and *ke* on the performance (the convergence of the accuracy rate, the cross-entropy loss, and the running time) of the deep learning model are confirmed by performing multiple validations using different hyperparameter values and different architectures. The value range of each parameter when the model reaches its relatively optimal state is obtained, which provides suggestions for the hyperparameter designs of deep learning network architectures.

3. The universal multiparameter mathematical correlation model gives some clarity to the process performed by the CNNs and their black box by mathematical derivation and experiment validation.

## 2. Related Work

### 2.1. The Interpretability of Deep CNNs

A deep learning model is called a “black box” because of its opaque learning and prediction processes, and the black box problem of deep learning has become a hot and difficult issue in artificial intelligence (AI) research [19,20,21,22,23]. Many methods have been proposed to explain deep learning models. For example, the local interpretable model-agnostic explanation (LIME) [24] is a method that is often used to simulate the output of a black box model with the advantages of form simplicity, easy modeling, and good interpretability, but it has difficulty dealing with models with complex features. The decision tree model [25] is a machine-learning-based self-explanatory model with a tree structure composed of internal nodes and leaf nodes, and it can quantify and explain the prediction logic of a CNN at the semantic level. Category activation approaches [26] based on the decision tree method have difficulty providing an understanding of the decision rules of trees as the tree depth and number of leaves increase. Class activation mapping (CAM) [27] is a method that can explain CNNs and identify the positions of classified objects. However, the CAM technique has a high model training cost, which greatly limits the application scenarios of the model. To solve the problems of the CAM method, the Grad-CAM [28] method was proposed, which is suitable for a variety of CNN models, and does not change the network structure or require retraining. Grad-CAM can generate class activation heat maps for a network on the basis of maintaining the original network structure, but cannot solve the gradient saturation problem. For this reason, the integrated gradient method [29] and deep-lift method [30] were proposed. Although these methods can explain a deep learning model to a certain extent or from different perspectives, they do not systematically explain the influences of hyperparameters (i.e., the learning rate, batch size, dropout rate, and convolution kernel size) on the deep learning performance of the model in principle.

### 2.2. The Optimizer of the Deep Convolution Neural Network Model Refers to Key Hyperparameters

Gradient methods (such as the gradient descent method (GD) [31], stochastic gradient descent method (SGD) [32], and small-batch stochastic gradient descent method (M-BGD) [33]) are the most commonly used optimization algorithms for deep learning models, and many subsequent algorithms have been developed accordingly to expand such methods. For example, the momentum method [34] is a new method for SGD to accelerate and suppress the correlation direction; this approach has the advantages of increasing the stability and speeding up the learning process of SGD, and the disadvantage is that the inertia of the system oscillates near the optimal solution when the learning speed is fixed. The Nesterov accelerated gradient (NAG) method [35,36] is an improvement of the momentum method that takes the cumulative adjustment of momentum into account, avoids moving too fast, and simultaneously improves the sensitivity of the developed model. Adaptive gradient (AdaGrad) [37] is an optimization algorithm that can adaptively adjust the learning rate of a model according to its gradient during the training process after setting the initial learning rate, but the disadvantage of this method is that as the cumulative squared gradient sum increases during the training period, the learning rate continues to shrink and eventually becomes infinitely small, which is not conducive to approaching the optimal solution in the later stage of training. Adaptive delta (AdaDelta) and root-mean-square propagation (RMSProp) were proposed to solve the problem of the AdaGrad learning rate monotonically decreasing [38]. AdaDelta uses the root-mean-square error of a given parameter to approximate the update process; RMSProp divides the learning rate by the exponentially decayed average of the squared gradient, then uses the learning rate and associates it with the gradient. They both use an attenuation coefficient to control the amount of gradient information considered, and may encounter saddle points during the training process. For the saddle point problem encountered during training, evolutionary stochastic gradient descent (ESGD) [39] was proposed for designing a more suitable adaptive learning rate method using a Hessian negative eigenvalue, and the convergence of this method was determined to be the same or faster than that of RMSProp. Adaptive moment estimation (Adam) [40] is another method for the adaptive calculation and adjustment of hyperparameters. This method not only stores the exponential decay average of the past gradient squares, but is also suitable for nonconvex optimization, noisy data, and sparse gradient problems in high-dimensional spaces. Adaptive max pooling (AdaMax) limits the upper bound of the learning rate and does not consider noise deviation; its parameter update method is simpler than that of Adam. Nesterov-accelerated adaptive moment estimation (Nadam) [41] is an optimization method that combines the Adam and NAG algorithms. The AdaptAhead [42] method is based on the Nesterov and RMSProp methods; it introduces first- and second-order attenuation rate parameters, initializes the model weights, and has a faster convergence speed than those of the methods on which it is based. In addition, in [43], an optimizer that combined dropout and SGD was used to improve the CNN algorithm, thereby improving the feature recognition rate and reducing the time cost of the examined CNN. In summary, hyperparameters such as *lr* and *m* are closely related to optimization algorithms. In addition, the hyperparameters of a deep learning model include not only the hyperparameters related to the corresponding optimization algorithm, but also the hyperparameters of the network structure, such as the size of the convolution kernel and the selection of the regularization method [44]. At present, these hyperparameters are usually selected based on experiments and previous experience. Optimizing hyperparameters is still the main problem with respect to training deep learning models. Therefore, studying the correlations between various hyperparameters and establishing a universal mathematical model is of great significance regarding the construction of deep learning models.

### 2.3. Commonly Used Hyperparameters

The *q*, *m*, *lr*, and *ke* are important parameters of the deep learning model, and their design and settings directly affect the performance of the deep learning model. The use of *q* can adjust the neural network being trained, and these random modifications to the network structure are thought to avoid neuronal coadaptation by making it impossible for two consecutive neurons to be completely interdependent [45,46,47]. The selection of *m* affects the quality of confidence interval estimators, and there is a certain relationship between sample size and optimal batch; a small batch is used to approximate the gradient of the loss function relative to the parameter [48]. The training is carried out in steps, and we consider a small batch in each step. An *lr* that is too small will cause the training algorithm to converge slowly, while an *lr* that is too large will cause the training algorithm to diverge [32]. Researchers [35] found that blindly superimposing the number of network layers is not an effective way to design an efficient and deeper network. The problem of designing a deep CNN under certain restricted conditions can be transformed into an optimization problem under restricted conditions, which is important. One of the influencing factors is the *ke*. By decomposing the convolution with an appropriate *ke*, more decoupling parameters can be obtained, and training can be accelerated. However, the current research only focuses on the experimental verification of a single parameter or two parameters. Based on this, we systematically described the impact of the four parameters of *q*, *m*, *lr*, and *ke* on the deep learning model; derived a mathematical formula for the relationship between the weights and bias of the four hyperparameters and the loss function of the deep learning model; and then verified the relative optimal value range of each hyperparameter through experimental verification.

## 3. Mathematical Deduction of the Model Optimization Process Considering a Single Hyperparameter

### 3.1. Basic Principles of a Deep Neural Network

A deep neural network is a neural network composed of many hidden layers, and its basic structure consists of an input layer, an output layer, and multiple hidden layers. Therefore, a deep neural network can explain the relationship between the input and output of its hidden layer by a linear model, and the input of the latter layer of the neural network is the output of the previous layer.

We make the following two assumptions: (1) The training dataset is a dataset *X* containing K categories, where a single sample *X* is an eigenvector with *i* neurons, $\vec{x}=\left(x_{1},x_{2},\cdots x_{i}\right)$ and (2) the deep learning model has L hidden layers, denoted as l=(1,2,…,L), $z^{\left(l\right)}$ represents the input vector of the *l* layer, and $y^{\left(l\right)}$ represents the output vector from the *l* layer (where $y^{\left(0\right)}=x$ is the original input).

Therefore, when the input is a single sample from a given category, the model input function and output function of each layer in the deep neural network are related to the weight and bias relations in the network, as shown in Equation (1):

(1) $$\left\{\begin{array}{l} y_{}^{\left(0\right)}=z_{}^{\left(0\right)}=\vec{x}\\ y_{}^{\left(1\right)}=f\left(z_{}^{\left(1\right)}\right)=f\left(\underset{i=1}{\sum }w_{i}^{\left(1\right)}\cdot x_{i}^{\left(1\right)}+b_{i}^{\left(1\right)}\right)=f\left(\underset{i=1}{\sum }w_{i}^{\left(1\right)}\cdot y_{}^{\left(0\right)}+b_{i}^{\left(1\right)}\right)\\ y_{}^{\left(2\right)}=f\left(z_{}^{\left(2\right)}\right)=f\left(\underset{i=1}{\sum }w_{i}^{\left(2\right)}\cdot x_{i}^{\left(2\right)}+b_{i}^{\left(2\right)}\right)=f\left(\underset{i=1}{\sum }w_{i}^{\left(2\right)}\cdot y_{}^{\left(1\right)}+b_{i}^{\left(2\right)}\right)\\ \;\;\;\;\;\;\;\;\;\;\;\;\;\;\;\;\;\;\;\;\;\;\;\;\;\;\;\;\;\;\;\;\;.\\ \;\;\;\;\;\;\;\;\;\;\;\;\;\;\;\;\;\;\;\;\;\;\;\;\;\;\;\;\;\;\;\;\;.\\ \;\;\;\;\;\;\;\;\;\;\;\;\;\;\;\;\;\;\;\;\;\;\;\;\;\;\;\;\;\;\;\;\;.\\ y_{}^{\left(l\right)}=f\left(z_{}^{\left(l\right)}\right)=f\left(\underset{i=1}{\sum }w_{i}^{\left(l\right)}\cdot x_{i}^{\left(l\right)}+b_{i}^{\left(l\right)}\right)=f\left(\underset{i=1}{\sum }w_{i}^{\left(l\right)}\cdot y_{}^{\left(l-1\right)}+b_{i}^{\left(l\right)}\right)\\ y_{}^{\left(l+1\right)}=f\left(z_{}^{\left(l+1\right)}\right)=f\left(\underset{i=1}{\sum }w_{i}^{\left(l+1\right)}\cdot x_{i}^{\left(l+1\right)}+b_{i}^{\left(l+1\right)}\right)=f\left(\underset{i=1}{\sum }w_{i}^{\left(l+1\right)}\cdot y_{}^{\left(l\right)}+b_{i}^{\left(l+1\right)}\right) \end{array}\right.$$

where $x_{i}^{\left(l\right)}$ represents the input of the l layer; $w_{i}^{\left(l\right)}$ and $b_{i}^{\left(l\right)}$ are the weight and bias of the l layer of $x_{i}^{\left(l\right)}$; respectively, and $f\left(Z\right)$ represents the activation function.

Then, when the input is a single sample of *K* categories, the input function and output function of each layer of the deep neural network are expressed as Equation (2):

(2) $$\left\{\begin{array}{l} y_{k}^{\left(0\right)}=z_{k}^{\left(0\right)}={\vec{x}}_{}\\ y_{k}^{\left(1\right)}=f\left(z_{k}^{\left(1\right)}\right)=f\left(\underset{i=1}{\sum }w_{k,i}^{\left(l\right)}\cdot x_{k,i}^{\left(1\right)}+b_{k,i}^{\left(1\right)}\right)=f\left(\underset{i=1}{\sum }w_{k,i}^{\left(1\right)}\cdot y_{k}^{\left(0\right)}+b_{k,i}^{\left(1\right)}\right)\\ y_{k}^{\left(2\right)}=f\left(z_{k}^{\left(2\right)}\right)=f\left(\underset{i=1}{\sum }w_{k,i}^{\left(2\right)}\cdot x_{k,i}^{\left(2\right)}+b_{k,i}^{\left(2\right)}\right)=f\left(\underset{i=1}{\sum }w_{k,i}^{\left(2\right)}\cdot y_{k}^{\left(1\right)}+b_{k,i}^{\left(2\right)}\right)\\ \;\;\;\;\;\;\;\;\;\;\;\;\;\;\;\;\;\;\;\;\;\;\;\;\;\;\;\;\;\;\;\;\;\;.\\ \;\;\;\;\;\;\;\;\;\;\;\;\;\;\;\;\;\;\;\;\;\;\;\;\;\;\;\;\;\;\;\;\;\;.\\ \;\;\;\;\;\;\;\;\;\;\;\;\;\;\;\;\;\;\;\;\;\;\;\;\;\;\;\;\;\;\;\;\;\;.\\ y_{k}^{\left(l\right)}=f\left(z_{k}^{\left(l\right)}\right)=f\left(\underset{i=1}{\sum }w_{k,i}^{\left(l\right)}\cdot x_{k,i}^{\left(l\right)}+b_{k,i}^{\left(l\right)}\right)=f\left(\underset{i=1}{\sum }w_{k,i}^{\left(l\right)}\cdot y_{k}^{\left(l-1\right)}+b_{k,i}^{\left(l\right)}\right)\\ y_{k}^{\left(l+1\right)}=f\left(z_{k}^{\left(l+1\right)}\right)=f\left(\underset{i=1}{\sum }w_{k,i}^{\left(l+1\right)}\cdot x_{k,i}^{\left(l+1\right)}+b_{k,i}^{\left(l+1\right)}\right)=f\left(\underset{i=1}{\sum }w_{k,i}^{\left(l+1\right)}\cdot y_{k}^{\left(l\right)}+b_{k,i}^{\left(l+1\right)}\right) \end{array}\right.$$

where $x_{k,i}^{\left(l\right)}$ represents the input sample from the k category in the l layer; $w_{k,i}^{\left(l\right)}$ and $b_{k,i}^{\left(l\right)}$ are the weight and the bias of the l layer of class k, respectively; and $f\left(Z\right)$ represents the activation function.

It can be seen from Equations (1) and (2) that in deep learning, bias and weight have decisive influences on the performance of each layer of the deep neural network model, regardless of the model input or output, when activation functions have been selected. Hyperparameters such as the learning rate, batch size, kernel size, and dropout rate affect the update processes of the network weights and biases. Therefore, this section mainly focuses on a correlation analysis regarding the influence of four hyperparameters, the kernel size, dropout rate, batch size, and learning rate, on the performance of a deep learning model.

### 3.2. Considering the Convolution Kernel Size

A CNN is a deep neural network using a convolutional layer. To extract data features (or carry out feature learning), the weight and bias of the network are shared for neurons in the same layer. Research [49] shows that the following factors determine the size of the output feature map after convolution: the input feature map size, the convolution kernel size, the stride size, and the padding size. The relationship between the output size and determinants is shown in Equation (3):

(3) $$O=\frac{I-ke+2P}{S}+1$$

In Equation (3), I is the size of the input image, S is the stride size, P is the padding size, and O is the output image size.

Equation (3) shows that the convolution kernel size is one of the main parameters affecting the size of the output feature map of each convolution layer.

When other parameters remain unchanged, the convolution kernel size not only affects the output image size of the convolutional layer, but also affects the number of weights and the number of total parameters of the convolutional layer. At this time, the relationship between the number of parameters of the convolutional layer and the convolutional kernel size can be expressed by Equation (4):

(4) $$\left\{\begin{array}{l} w_{C}=ke^{2}\times ch\times N\\ B_{C}=N\\ P_{C}=W_{C}-B_{C} \end{array}\right.$$

where $W_{C}$ and $B_{C}$ are the numbers of weights and biases of the convolutional layer, *Pc* is the total number of parameters of the convolutional layer, *N* is the predefined number of cores, and ch is the number of input image channels.

At the same time, the size of the convolution kernel also affects the number of output parameters of the fully connected layer. Since the fully connected layer has a characteristic that the length of its output vector is equal to the number of neurons in the layer, the number of parameters is the sum of all weights and biases. The fully connected layer is divided into two cases; namely, the fully connected layer of the previous layer connected to the convolutional layer and the fully connected layer of the whole layer connected to the fully connected layer. The number of parameters is calculated as follows for the two cases:

(a) The calculation process for the parameters of the fully connected layer connected to the convolutional layer is shown in Equation (5):

(5) $$\left\{\begin{array}{l} W_{cf}=O^{’2}\times N\times F\\ B_{cf}=F\\ P_{cf}=W_{cf}-B_{cf} \end{array}\right.$$

where $W_{cf}$ and $B_{cf}$ are the numbers of weights and biases of the fully connected layer connected to the convolutional layer, respectively; $O^{’}$ is the output image size of the previous convolutional layer; F is the number of neurons in the current fully connected layer; and $P_{cf}$ is the total number of parameters in the current fully connected layer.

(b) The calculation process for the parameters of the fully connected layer connected to another fully connected layer is shown in Equation (6):

(6) $$\left\{\begin{array}{l} W_{ff}=F_{-1}\times F\\ B_{ff}=F\\ P_{ff}=W_{ff}-B_{ff} \end{array}\right.$$

where $W_{ff}$ and $B_{ff}$ are the numbers of weights and biases of the current fully connected layer, respectively; $F_{-1}$ is the number of neurons in the previous fully connected layer; and $P_{ff}$ is the total number of parameters in the current fully connected layer.

The convolutional kernel size is an important parameter for a CNN that affects the number of parameters involved in the calculation of the neural network.

### 3.3. Considering the Dropout Rate

Dropout is proposed to solve the overfitting problem of complex feedforward neural networks when training small datasets, and the dropout rate is used as a hyperparameter for training deep neural networks. The principle of dropout is to temporarily discard the neural network unit from the network in accordance with a certain probability during the training process of the deep learning network [50,51].

For *l* layer, r is a vector of independent Bernoulli random variables that obeys the Bernoulli distribution:

(7) $$\left\{\begin{array}{l} P_{r}(r^{\left(l\right)}=1)=1-q\\ P_{r}(r^{\left(l\right)}=0)=q \end{array}\right.$$

It is assumed that dropout is adopted to discard the neurons in the neural network with a probability of *q*. Suppose that the l layer adopts the neural network model of dropout; then, the mathematical expressions of the output and input after refinement with dropout are shown in Equation (8):

(8) $$\left\{\begin{array}{l} {\tilde{y}}_{}{}^{\left(l\right)}=r^{\left(l\right)}*y_{}{}^{\left(l\right)}\\ z_{}{}^{\left(l+1\right)}=w_{i}^{(}{}^{l+1)}{\tilde{y}}_{}{}^{l}+b_{i}^{(}{}^{l+1)}=w_{i}^{(}{}^{l+1)}r^{\left(l\right)}*y_{}{}^{\left(l\right)}+b_{i}^{(}{}^{l+1)}\\ y_{}^{(}{}^{l+1)}=f(z_{}^{(}{}^{l+1)}) \end{array}\right.$$

For a sample from a dataset with k categories, according to Equation (4), the output of the corresponding layer is:

(9) $$\left\{\begin{array}{l} {\tilde{y}}_{k}{}^{\left(l\right)}=r^{\left(l\right)}*y_{k}{}^{\left(l\right)}\\ \begin{array}{l} z_{k}{}^{\left(l+1\right)}=w_{k,i}^{\left(l+1\right)}{\tilde{y}}_{k}^{\left(l\right)}+b_{k,i}^{\left(l+1\right)}=w_{k,i}^{\left(l+1\right)}r^{\left(l\right)}*y_{k}{}^{\left(l\right)}+b_{k,i}^{\left(l+1\right)}\\ y_{k}^{(}{}^{l+1)}=f(z_{k}^{(l+1}{}^{)}) \end{array} \end{array}\right.$$

where ${\hat{y}}^{\left(l\right)}$ is the vector output from the layer after being refined by dropout.

However, in the testing phase, the weight is scaled; that is, the neural network obtained during the test is used without dropout.

### 3.4. Correlation Analysis Regarding the Batch Size and Learning Rate in the Binary and Multiclassification Case

The learning rate and batch size directly determine the weight update of the deep learning model, and affect the most important parameters with respect to model performance (convergence) [52,53]. The deep learning model process mainly includes convolution, dropout processing, a fully connected layer, and finally a classification model function for achieving target classification. According to the given sample label categories, we can divide the target classification problem into binary classification (the sample label has only two categories) and multiclass classification (the sample label has more than two categories). According to the target classification situation, the applicable classification model function is different.

Binary classification divides a sample into two categories (Class 1 and Class 2). Assuming that Class 1 is a positive sample, the probability that the sample is of the positive type is expressed as the probability of the sample belonging to Class 2: $1-P\left(C_{1}|x\right)$, and the classification model function is based on the sigmoid function. That is, the activation function f at this time is the sigmoid function. In a multilayer CNN model with an L-type hidden layer, the classification link is located in the output layer, and the input and output mathematical expressions are shown in Equation (10):

(10) $$\left\{\begin{array}{l} z_{}{}^{\left(l+1\right)}=\overset{}{\underset{i=1}{\sum }}w_{i}{}^{\left(l+1\right)}\cdot x_{i}^{\left(l+1\right)}+b_{i}^{\left(l+1\right)}\\ f(z_{}^{\left(l+1\right)})=P(C_{1}|x)\\ f(z_{}{}^{\left(l+1\right)})=\sigma (z_{}{}^{\left(l+1\right)})=\frac{1}{1+e^{-}{}^{z_{}{}^{\left(l+1\right)}}} \end{array}\right.$$

where $P\left(C_{1}|x\right)$ represents the probability of a positive sample, and $P\left(C_{1}|x\right)\epsilon \left(0,1\right)$.

According to the likelihood estimation and the chain rule, when the type of sample label is positive (when $P\left(C_{1}|x\right)=1$), the loss function is 0. If the type of sample label is negative (when when $P\left(C_{1}|x\right)=0$), the loss function is 1. According to Equation (10), the loss function is a function of the network weight and bias, and the loss function can be used to find the best $w^{*}$ and $b^{*}$ for minimization purposes.

Let $f_{w,b}(x)=f(z_{}^{\left(l+1\right)})=P(C_{1}|x)$; for a single sample, its loss function can be expressed as:

(11) $$\mathrm{Cos}t\left(f_{w,b\;}\left(x\right),y\right)=\left\{\begin{array}{c} -\log\left(f_{w,b}\left(x\right)\right)\;\;\mathrm{if}\;\hat{y}=1\\ -\log\left(1-f_{w,b\;}\left(x\right)\right)\;\mathrm{if}\;\hat{y}=0 \end{array}\right.$$

In Equation (11),$\;\hat{y}$ is the target label, $\hat{y}=1$ represents the sample belonging to Class 1, and $\hat{y}=0$ represents the sample belonging to Class 2.

By writing the combined Equation (11) into a single equation, the equation of the cross-entropy loss function can be written as:

(12) $$\mathrm{Cos}t(f_{w,b}(x),\hat{y})=-[(\hat{y})\log(f_{w,b}(x))+(1-\hat{y})\log(1-f_{w,b}(x))]$$

For a sample with batch size = m, its loss function is the mean value of the loss function jointly caused by *x* and *y*:

(13) $$J(w,b)=\frac{1}{m}\overset{m}{\underset{j}{\sum }}\mathrm{Cos}t(f_{w,b}(x^{\left(j\right)}),{\hat{y}}^{\left(j\right)})$$

Furthermore, Equation (13) is substituted into Equation (12) to obtain:

(14) $$J(w,b)=-\frac{1}{m}\overset{m}{\underset{j=1}{\sum }}\left[({\hat{y}}^{\left(j\right)})\log(f_{w,b}(x^{\left(j\right)}))+(1-{\hat{y}}^{\left(j\right)})\log(1-f_{w,b}(x^{\left(j\right)}))\right]$$

According to Equation (10), Equation (14) can be written as:

(15) $$J(w,b)=-\frac{1}{m}\overset{m}{\underset{j=1}{\sum }}[{\hat{y}}^{\left(j\right)}\ln f_{w,b}(x^{\left(j\right)})+(1-{\hat{y}}^{\left(j\right)})\ln(1-f_{w,b}(x^{\left(j\right)}))]$$

The partial derivative of *J*(*w,b*) with respect to $w_{i}$ is calculated according to the gradient descent method and the chain rule:

(16) $$\begin{array}{ll} \frac{\partial J(w,b)}{\partial w_{i}} & =-\frac{1}{m}\overset{m}{\underset{j}{\sum }}\left[{\hat{y}}^{\left(j\right)}(1-f_{w,b}(x^{\left(j\right)}))x_{i}^{\left(j\right)}-(1-{\hat{y}}^{\left(j\right)})(1-f_{w,b}(x^{\left(j\right)}))x_{i}^{\left(j\right)}\right)\\ & =-\frac{1}{m}\overset{m}{\underset{j}{\sum }}[{\hat{y}}_{}{}^{\left(j\right)}-{\hat{y}}^{\left(j\right)}f_{w,b}(x^{\left(j\right)})-f_{w,b}(x^{\left(j\right)})+{\hat{y}}^{\left(j\right)}f_{w,b}(x^{\left(j\right)})]x_{i}^{\left(j\right)}\\ & =-\frac{1}{m}\overset{m}{\underset{j}{\sum }}\left({\hat{y}}^{\left(j\right)}-f_{w,b}(x^{\left(j\right)})\right)x_{i}^{\left(j\right)} \end{array}$$

According to the gradient descent method, the update iteration expression of $w_{i}$ is:

(17) $$w_{i}{}^{\left(l+1\right)}=w_{i}{}^{\left(l+1\right)}-lr\frac{\partial J(w,b)}{\partial w_{i}}=w_{i}{}^{\left(l+1\right)}-\frac{lr}{m}\overset{m}{\underset{j}{\sum }}\left({\hat{y}}^{\left(j\right)}-f_{w,b}(x^{\left(j\right)})\right)x_{i}^{\left(l+1,j\right)}$$

Similarly, the update iteration expression of $b_{i}$ is:

(18) $$b_{i}{}^{\left(l+1\right)}=b_{i}{}^{\left(l+1\right)}-lr\frac{\partial J(w,b)}{\partial b_{i}}=b_{i}{}^{\left(l+1\right)}-\frac{lr}{m}\overset{m}{\underset{j}{\sum }}\left({\hat{y}}^{\left(j\right)}-f_{w,b}(x^{\left(j\right)})\right)$$

where *lr* represents the learning rate.

The softmax function is often used as a multiclassification activation function in deep learning models, as it is an extension of logistic regression. The features of this function are that the model has multiple outputs and that the number of outputs equals the number of categories. The output value is the probability of the input sample *X* belonging to each category. Finally, the highest probability denotes that the sample is predicted to belong to the corresponding category. For a learning model has L hidden layers that uses the softmax function as the activation function of the output layer, its mathematical description is as follows:

(19) $$\left\{\begin{array}{l} \;\;\;\;\;\;\;\;\;\;\;\;\;\;\;\;\;z_{k}^{\left(l+1\right)}{=\vec{w}}_{k}^{\left(l+1\right)}\cdot {\vec{x}}_{}{}^{\left(l+1\right)}{+b}_{k}^{\left(l+1\right)}\\ f_{w,b}\left(\vec{x}\right)=\frac{e^{z_{k}^{\left(l+1\right)}}}{\sum_{n=1}^{k}e^{z_{n}^{\left(l+1\right)}}}=\frac{e^{{\vec{w}}_{k}^{\left(l+1\right)}\cdot {\vec{x}}_{}^{\left(l+1\right)}+b_{k}^{\left(l+1\right)}}}{\sum_{n=1}^{k}e^{{\vec{w}}_{n}^{\left(l+1\right)}\cdot {\vec{x}}_{}^{\left(l+1\right)}+b_{n}^{\left(l+1\right)}}} \end{array}\right.$$

For a sample with a batch size m, the target is to learn through the model, so the target loss function (also known as the logarithmic likelihood cost function) is:

(20) $$J(w,b)=-\frac{1}{m}\overset{m}{\underset{j=1}{\sum }}\overset{K}{\underset{k=1}{\sum }}A\left\{{\hat{y}}^{\left(j\right)}=k\right\}\log(\frac{e^{{\vec{w}}_{k}^{\left(l+1,j\right)}\cdot {\vec{x}}_{}{}^{\left(l+1,j\right)}+b_{k}^{\left(l+1\right)}}}{\overset{K}{\underset{n=1}{\sum }}e^{{\vec{w}}_{n}^{\left(l+1,j\right)}\cdot {\vec{x}}_{}{}^{\left(j\right)}+b_{n}^{\left(l+1\right)}}})$$

where $A\left\{\cdot \right\}$ is the indicator function, which means that when ${\hat{y}}^{\left(j\right)}=k$ is true, the function value is 1; otherwise, the function value is 0. According to binary classification, the logarithmic cost function is transformed into the cross-entropy loss function to obtain the learning objective loss function, as shown in Equation (21):

(21) $$j(w,b)=-\frac{1}{m}\overset{m}{\underset{j=1}{\sum }}\overset{k}{\underset{k=1}{\sum }}\left(A\left\{{\hat{y}}^{\left(j\right)}=k\right\}{\log(e}^{{\vec{w}}_{k}^{(l+1)}\cdot {\vec{x}}^{(l+1,j)}{+b}_{k}^{\left(l+1\right)}}\right)-1\left\{{\hat{y}}^{\left(j\right)}=k\right\}\log(\overset{k}{\underset{n=1}{\sum }}e^{{\vec{w}}_{n}^{\left(l+1\right)}\cdot {\vec{x}}^{(l+1,j)}{+b}_{n}^{\left(l+1\right)}}))$$

Equation (21) can be simplified as:

(22) $$J(w,b)=-\frac{1}{m}\overset{m}{\underset{j=1}{\sum }}(\overset{K}{\underset{k=1}{\sum }}\left(A\left\{{\hat{y}}^{\left(j\right)}=k\right\}\log e^{{\vec{w}}_{k}^{\left(l+1\right)}\cdot {\vec{x}}^{\left(l+1)j\right)}+b_{k}^{\left(l+1\right)}}\right)-\log(\overset{K}{\underset{n=1}{\sum }}e^{{\vec{w}}_{n}^{\left(l+1\right)}\cdot {\vec{x}}^{\left(l+1,j\right)}+b_{n}^{\left(l+1\right)}}))$$

According to the gradient descent minimization loss function and the chain rule, the partial derivative of J(w,b) is calculated with respect to ${\vec{w}}_{k}$.

(23) $$\frac{\partial J(w,b)}{\partial {\vec{w}}_{k}^{\left(l+1\right)}}=-\frac{1}{m}\overset{m}{\underset{j=1}{\sum }}(\overset{K}{\underset{k=1}{\sum }}(A\left\{{\hat{y}}^{\left(j\right)}=k\right\}\frac{e^{{\vec{w}}_{k}^{\left(l+1\right)}\cdot {\vec{x}}^{\left(l+1,j\right)}+b_{k}^{\left(l+1\right)}}*{\vec{x}}^{\left(l+1,j\right)}}{e^{{\vec{w}}_{k}^{\left(l+1\right)}\cdot {\vec{x}}^{\left(l+1,j\right)}+b_{k}^{\left(l+1\right)}}}-\frac{e^{{\vec{w}}_{k}^{\left(l+1\right)}\cdot {\vec{x}}^{\left(l+1,j\right)}+b_{{}_{k}}^{\left(l+1\right)}}*{\vec{x}}^{\left(l+1,j\right)}}{\overset{K}{\underset{n=1}{\sum }}e^{{\vec{w}}_{n}^{\left(l+1\right)}\cdot {\vec{x}}^{\left(l+1,j\right)}+b_{n}^{\left(l+1\right)}}})$$

Equation (23) is simplified as follows:

(24) $$\begin{array}{ll} \frac{\partial J(w,b)}{\partial {\vec{w}}_{k}^{\left(l+1\right)}} & =-\frac{1}{m}\overset{m}{\underset{j=1}{\sum }}(\overset{K}{\underset{k=1}{\sum }}(A\left\{{\hat{y}}^{\left(j\right)}=k\right\}{\vec{x}}^{\left(l+1,j\right)}-P({\hat{y}}^{\left(j\right)}=k\left|x^{\left(l+1,j\right)}\right.)*{\vec{x}}^{\left(l+1,j\right)})\\ & =-\frac{1}{m}\overset{m}{\underset{j=1}{\sum }}({\vec{x}}^{\left(l+1,j\right)}-P({\hat{y}}^{\left(j\right)}=k\left|x^{\left(l+1,j\right)}\right.)*{\vec{x}}^{\left(l+1,j\right)}) \end{array}$$

According to the gradient descent method, the update iteration expression of ${\vec{w}}_{k}$ is:

(25) $${\vec{w}}_{k}^{\left(l+1\right)}={\vec{w}}_{k}^{\left(l+1\right)}-lr\frac{\partial J(w,b)}{\partial w_{k}}={\vec{w}}_{k}^{\left(l+1\right)}-\frac{lr}{m}\overset{m}{\underset{j=1}{\sum }}({\vec{x}}^{\left(l+1,j\right)}-P({\hat{y}}^{\left(j\right)}=k\left|x^{\left(l+1,j\right)}\right.)*{\vec{x}}^{\left(l+1,j\right)})$$

Through the gradient descent method, the iterative expression of $b_{k}$ can also be obtained as follows:

(26) $$b_{k}^{\left(l+1\right)}=b_{k}^{\left(l+1\right)}-lr\frac{\partial J(w,b)}{\partial b_{k}}{=b}_{k}^{\left(l+1\right)}-\frac{lr}{m}\overset{m}{\underset{j=1}{\sum }}(1-P(y^{\left(j\right)}=k|{\vec{x}}^{\left(l+1,j\right)}))$$

Through the above analysis, the learning rate and batch size directly determine the updating of the weight and bias of the deep learning model, and there is a correlation between them, as shown in Equations (17), (18), (25), and (26). The learning rate and batch size are important parameters that affect the performance of a deep learning model.

## 4. Proposed Generalized Mathematical Derivation Considering Multiple Parameters 

After the analysis in Section 3.2, Section 3.3, Section 3.4, it is known that the learning rate, dropout rate, batch size, and kernel size all impact the performance of a deep learning model. In particular, the learning rate and batch size directly affect the final loss function parameters of the deep learning model output layer (weight and bias). The number of update iterations and the optimal value ranges of the learning rate, and batch size can be found through the relationship between the two. Therefore, studying the correlations between multiple parameters is of great significance for our choice of hyperparameters.

According to Equations (7) and (8), it is assumed that dropout is adopted in the deep learning model layers, and the loss function parameter updates (Equations (17) and (18)) for the binary classification problem can be expressed as:

(27) $$\begin{array}{ll} w_{i}^{\left(l+1\right)} & =w_{i}^{\left(l+1\right)}-\frac{lr}{m}\overset{m}{\underset{j}{\sum }}({\hat{y}}^{\left(j\right)}-f_{w,b}(x_{}{}^{\left(j\right)}))x_{i}^{\left(l+1,j\right)}\\ & =w_{i}^{\left(l+1\right)}-\frac{lr}{m}\overset{m}{\underset{j}{\sum }}({\hat{y}}^{\left(j\right)}-f_{w,b}(\underset{i}{\sum }w_{i}^{(}{}^{l+1)}{\tilde{y}}_{i}{}^{l}+b_{i}^{(}{}^{l+1)}))x_{i}^{\left(l+1,j\right)}\\ & =w_{i}^{\left(l+1\right)}-\frac{lr}{m}\overset{m}{\underset{j}{\sum }}({\hat{y}}^{\left(j\right)}-f(\underset{i}{\sum }w_{i}^{(}{}^{l+1)}r^{\left(l\right)}\;*y_{i}{}^{\left(l\right)}+b_{i}^{(}{}^{l+1)})x_{i}^{\left(l+1,j\right)}\\ & =w_{i}^{\left(l+1\right)}-\frac{lr}{m}\overset{m}{\underset{j}{\sum }}({\hat{y}}^{\left(j\right)}-f(\underset{i}{\sum }w_{i}^{(}{}^{l+1)}r^{\begin{array}{c} \left(l\right) \end{array}}\;\;*f(\underset{i}{\sum }w_{i}^{(}{}^{l)}*y_{i}{}^{\left(l\right)}+b_{i}^{(}{}^{l)})+b_{i}^{(}{}^{l+1)})x_{i}^{\left(l+1,j\right)} \end{array}$$

(28) $$\begin{array}{ll} b_{i}^{\left(l+1\right)} & =b_{i}^{\left(l+1\right)}-\frac{lr}{m}\overset{m}{\underset{j}{\sum }}({\hat{y}}^{\left(j\right)}-f_{w,b}(x_{}^{\left(j\right)}))\\ & =b_{i}^{\left(l+1\right)}-\frac{lr}{m}\overset{m}{\underset{j}{\sum }}({\hat{y}}^{\left(j\right)}-f_{w,b}(\underset{i}{\sum }w_{i}^{(}{}^{l+1)}{\tilde{y}}_{i}{}^{l}+b_{i}^{(}{}^{l+1)}))\\ & =b_{i}^{\left(l+1\right)}-\frac{lr}{m}\overset{m}{\underset{j}{\sum }}({\hat{y}}^{\left(j\right)}-f_{w,b}(\underset{i=1}{\sum }w_{i}^{(}{}^{l+1)}r_{}^{(}{}^{l)}\;*y_{i}{}^{\left(l\right)}+b_{i}^{(}{}^{l+1)}))\\ & =b_{i}^{\left(l+1\right)}-\frac{lr}{m}\overset{m}{\underset{j}{\sum }}({\hat{y}}^{\left(j\right)}-f_{w,b}(\underset{i=1}{\sum }w_{i}^{(}{}^{l+1)}r_{}^{(}{}^{l)}\;*f(\underset{i=1}{\sum }w_{i}^{(}{}^{l)}y_{i}{}^{\left(l\right)}+b_{i}^{\left(l\right)})+b_{i}^{(}{}^{l+1)})) \end{array}$$

In a multiclassification problem, it is assumed that the dropout model is used in layer *l* of the deep learning model; according to $\frac{e^{{\vec{w}}_{k}^{\left(l+1\right)}\cdot {\vec{x}}^{\left(l+1\right)}+b_{k}}}{\sum_{n=1}^{K}e^{{\vec{w}}_{n}^{\left(l+1\right)}\cdot {\vec{x}}^{\left(l+1\right)}+b_{n}^{\left(l+1\right)}}}=P(\hat{y}=k\left|\vec{x})\right.$ and Equation (8), the parameter update iteration (Equation (27)) of the loss function (Equation (28)) can be expressed as:

(29) $$\begin{array}{ll} {\vec{w}}_{k}{}^{\left(l+1\right)} & ={\vec{w}}_{k}{}^{\left(l+1,j\right)}-lr\frac{\partial J(w,b)}{\partial w_{k}}={\vec{w}}_{k}-\frac{lr}{m}\overset{m}{\underset{j=1}{\sum }}({\vec{x}}^{\left(j\right)}-P(y^{\left(k\right)}=k\left|x^{(l+1,}{}^{j)}\right.)*{\vec{x}}^{(l+1,}{}^{j)})\\ & ={\vec{w}}_{k}-\frac{lr}{m}\overset{m}{\underset{j=1}{\sum }}(({\vec{x}}^{\left(j\right)}-\frac{e^{{\vec{w}}_{k}^{\left(l+1\right)}\cdot \vec{x}+b_{k}}}{\sum_{n=1}^{K}e^{{\vec{w}}_{n}^{\left(l+1\right)}\cdot \vec{x}+b_{n}^{\left(l+1\right)}}})*{\vec{x}}^{(l+1,}{}^{j)})\\ & ={\vec{w}}_{k}-\frac{lr}{m}\overset{m}{\underset{j=1}{\sum }}(({\vec{x}}_{}{{}^{(l+1,}}^{j)}-f(\underset{i}{\sum }{\vec{w}}_{k,i}^{\left(l+1\right)}\;\cdot x_{i}{}^{\left(l+1,j\right)}{+b}_{k,i}^{\left(l+1\right)})*{\vec{x}}_{}{}^{(l+1{,}^{j)}})\\ & ={\vec{w}}_{k}-\frac{lr}{m}\overset{m}{\underset{j=1}{\sum }}(({\vec{x}}_{}{{}^{(l+1,}}^{j)}-f(\underset{i}{\sum }{\vec{w}}_{k,i}^{\left(l+1\right)}r_{}^{(}{}^{l)}\;*y_{i}{}^{\left(l,j\right)}\;{+b}_{k,i}^{\left(l+1\right)})*{\vec{x}}_{}{}^{(l+1{,}^{j)}})\\ & ={\vec{w}}_{k}-\frac{lr}{m}\overset{m}{\underset{j=1}{\sum }}(({\vec{x}}_{i}{{}^{(l+1,}}^{j)}-f(\underset{i=1}{\sum }w_{i}^{(}{}^{l+1)}r_{}^{(}{}^{l)}\;*f(\underset{i=1}{\sum }w_{i}^{(}{}^{l)}y_{i}{}^{\left(l\right)}+b_{i}^{\left(l\right)})+b_{k,i}^{\left(l+1\right)})*{\vec{x}}^{(l+1{,}^{j)}}) \end{array}$$

(30) $$\begin{array}{ll} b^{l+1}{}_{k} & =b^{\left(l+1\right)}{}_{k}-lr\frac{\partial Jw,b}{\partial w_{k}}=b_{k}-\frac{lr}{m}\overset{m}{\underset{j=1}{\sum }}({\vec{x}}^{\left(j\right)}-P(y^{\left(k\right)}=k\left|x^{(l+1,}{}^{j)}\right.))\\ & =b^{\left(l+1\right)}{}_{k}-\frac{lr}{m}\overset{m}{\underset{j=1}{\sum }}(({\vec{x}}^{\left(j\right)}-\frac{e^{{\vec{w}}_{k}^{\left(l+1\right)}\cdot \vec{x}+b_{k}^{\left(l+1\right)}}}{\sum_{n=1}^{K}e^{{\vec{w}}_{n}^{\left(l+1\right)}\cdot \vec{x}+b_{n}^{\left(l+1\right)}}}))\\ & =b^{\left(l+1\right)}{}_{k}-\frac{lr}{m}\overset{m}{\underset{j=1}{\sum }}((1-f(\underset{i}{\sum }{\vec{w}}_{k,i}^{\left(l+1\right)}\;\cdot x_{i}{}^{\left(l+1,j\right)}{+b}_{k,i}^{\left(l+1\right)}))\\ & =b^{\left(l+1\right)}{}_{k}-\frac{lr}{m}\overset{m}{\underset{j=1}{\sum }}((1-f(\underset{i}{\sum }{\vec{w}}_{k,i}^{\left(l+1\right)}r_{}^{(}{}^{l)}\;*y_{i}{}^{\left(l,j\right)}\;{+b}_{k,i}^{\left(l+1\right)}))\\ & =b^{\left(l+1\right)}{}_{k}-\frac{lr}{m}\overset{m}{\underset{j=1}{\sum }}(({\vec{x}}_{i}{{}^{(l+1,}}^{j)}-f(\underset{i=1}{\sum }w_{k,i}^{\left(l+1\right)}r_{}^{(}{}^{l)}\;*f(\underset{i=1}{\sum }w_{k,i}{}^{\left(l\right)}y_{i}{}^{\left(l\right)}+b_{i}^{\left(l\right)})+b_{k,i}^{\left(l+1\right)})) \end{array}$$

According to the above analysis, the dropout rate is also an important parameter affecting the performance of the deep learning model, and it has certain correlations with the learning rate and batch size, as shown in Equations (27)–(30). The range value for parameter optimization can be found through these correlation relationships.

Moreover, when we calculate the number of parameters used by the deep learning model according to the size of the convolution kernel, the number of parameters is also related to whether dropout is used.

If the convolution layer adopts dropout, according to Equation (4) and the dropout principle, the number of parameters involved in the calculation of the convolution layer is:

(31) $$\left\{\begin{array}{l} w_{c}=(1-q)ke^{2}\times ch\times N\\ B_{c}=N\\ P_{c}=(1-q)W_{c}-B_{c} \end{array}\right.$$

If the fully connected layer adopts dropout, the number of parameters of the fully connected layer connected to the convolutional layer according to Equation (5), and the dropout principle is calculated as shown in Equation (32):

(32) $$\left\{\begin{array}{l} w_{cf}=(1-q)ke^{2}\times ch\times N\\ B_{cf}=N\\ P_{cf}=(1-q)W_{cf}-B_{cf} \end{array}\right.$$

According to Equation (6) and the dropout principle, the calculation processes for all parameters connected to the fully connected layer are shown in Equation (33):

(33) $$\left\{\begin{array}{l} W_{ff}=(1-q)F_{-1}\times F\\ B_{ff}=F\\ P_{ff}=(1-q)W_{ff}-B_{ff} \end{array}\right.$$

Through Equations (32) and (33), we could calculate the number of parameters involved in the calculation of each layer of the deep neural network model when we used dropout, which helped us effectively understand the deep learning model.

## 5. Experiments and Analyses

After the correlation analysis in Section 4 we know that the four hyperparameters (the kernel size, learning rate, dropout rate and batch size) theoretically have an impact on the performance of the deep learning model. To better study the influence of each hyperparameter on model performance, in this section, we designed experiments according to the single-factor experimental method. In this section, we conducted hyperparameter experiments on the MNIST dataset [54]. The MNIST dataset is a publicly available dataset that has been adopted by many studies in image processing and has almost become a “paradigm”. The MNIST training data set contains 60,000 samples and the test data set contains 10,000 samples, and each image in the MNIST dataset consists of 28 × 28 pixel points, each represented by a grayscale value. To avoid randomness, the training accuracy was defined as the average value calculated after we repeated each parameter experiment three times. In these experiments, the adopted multilayer neural network architecture consisted of three convolution layers and three fully connected layers. In addition to using softmax as the classification function for the output layer, the activation function of the hidden layer was selected as a leaky rectified linear unit “Leaky-ReLU (LReLU)” [55]. The reason for choosing this network was that it is easy to train and sufficient for carrying out experiments on the performance of the hyperparameter-influenced learning model. In order to ensure the accuracy of the experimental results, 10 cross validation tests were carried out on LRelu, Relu [50], and scaled exponential linear unit (Selu) [51] models, respectively.

### 5.1. Experimental Environment

The Python programming language was used for coding a CNN learning algorithm, which was implemented on a 64-bit Ubuntu 18.04.5 LTS. TensorFlow 1.14.0 was installed to evaluate the performance of the designed model. The model performance was tested and analyzed under different *q*, *m*, *ke,* and *lr*, and the relative optimal parameters were obtained. Then, the optimal parameters were combined and verified. All results were tested on the same computer model, and the main configuration of the computer was as follows: an Intel (R) Core (TM) i7-9700F CPU @ 3.00 GHz, 8.00 GB of RAM, and an NVIDIA GeForce GTX 1660 GPU.

### 5.2. Experimental Design

We conducted two experiments: an experiment on the influence of a single parameter on the performance of the deep learning model, and an optimization combination verification experiment. The purpose of the former was to verify the influences of the *q*, *m*, *lr,* and *ke* on model performance, and the optimal value range of each parameter was obtained through experiments. On the basis of the former, the purpose of the latter was to combine the optimal range values of all parameters obtained from the former to further verify the influence of each parameter on the performance of the deep learning model. 

According to the first purpose, we designed experiments as presented in Section 5.4. The variables of our experiments were four hyperparameters: the dropout rate, batch size, learning rate, and kernel size. Each hyperparameter variable was valued at equal intervals, and the number of training steps was 100,000. According to the second purpose, we designed an experiment as shown in Section 5.5 with 100,000 training steps.

### 5.3. Hyperparameter Selection

Equations (27)–(33) prove that the hyperparameters (*q*, *m*, *ke*, *lr*) have an effect on the weight and bias of the model when other parameters are constant. They show that *m* and *lr* are important parameters affecting the updating of model weights. If *lr* is too large, the model does not converge; if *lr* is too small, the model converges very slowly or cannot be learned, and the size of *m* value affects the model convergence to a certain extent [52,53,56]. The value of m is often a power of 2 [57]. Equations (7)–(9) indicate that the selection of the q value is one of the keys to parameter tuning, and directly affects the robustness of the model. According to the principle of dropout and the rules of neural network parameter updating, when *m*, *lr,* and *ke* are constant values, the value of *q* ranges from {0.3,0.5} [43,45,56]. The *ke* value directly determines the parameters and computation required by the model. If the value is too small, the model’s receptive field is not enough to learn features; if the value is too large, the model’s parameters are too many to lead to computation, the *ke* value range is {3, 7} [49].

### 5.4. Experiment and Analysis Based on Single Parameter Optimization

(A) On the *q*

To study the effect of the dropout rate on the examined deep learning model, we set different settings as *q* = {0.1, 0.2, 0.3, 0.4, 0.5, 0.6, 0.7, 0.8, 0.9}, and the other three parameters were *m* = 64, *ke* = 5, and *lr* = 1 × 10^−4^.

**Figure 1:**
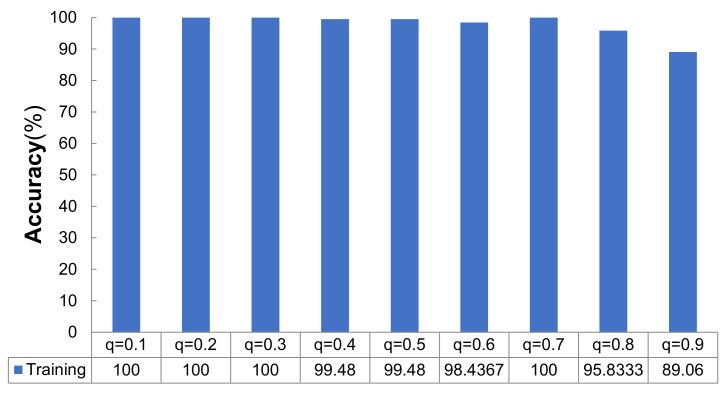
Training accuracies obtained under different *q* (%).

**Figure 2:**
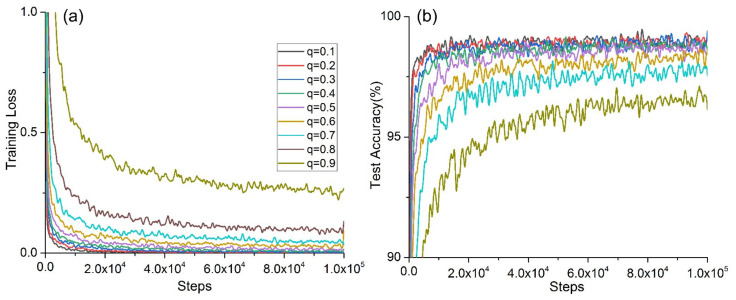
Model convergence under different *q*: (**a**) convergence of training cross-entropy loss; (**b**) convergence of test accuracy.

According to Figure 1, the training accuracy of the network model varied with different dropout rates as follows: when *q* = {0.1, 0.2, 0.3, 0.7}, the training accuracy was 100%; when *q* = {0.4, 0.5, 0.6, 0.8, 0.9}, the training accuracies were 99.48%, 99.48%, 98.44%, 95.83%, and 89.06%, respectively. It can be concluded that the average accuracy did not change when the dropout rate was between 0.1–0.3 in the training stage. Then, when *q* increased from 0.4 to 0.7, the accuracy range was very small, but when *q* increased from 0.7 to 0.9, the training accuracy decreased sharply. The convergence of the accuracy rate is shown in Figure 2. As shown in Figure 2a, the fastest convergence rate on the training set was achieved when *q* = {0.5, 0.4, 0.3}. Figure 2b shows that the convergence rate was relatively fast for the test set when *q* = {0.5, 0.4, 0.3}.

Table 1 shows that the variation in time consumption could be divided into three distinct stages: in the first stage, when *q* < 0.4, the time consumption increased with the increase in the dropout value; in the second stage, when 0.4 ≤ *q* ≤ 0.6, the time taken for *q* = 0.4 decreased relative to that required for *q* = 0.3, but when the dropout increases from 0.4 to 0.6, the time increased with the increase in the dropout value. Stage 3: when *q* = 0.6 increased to *q* = 0.8, the time decreased as the dropout value increased. However, when *q* = 0.8 and *q* = 0.9, there was no significant change in the time consumption of the operation.

Considering the convergence of the accuracy and cross-entropy loss values during model training, the convergence of the accuracy during testing and the time consumption incurred when going through the same steps, the experiment showed that the best performance obtained by the deep neural network model using dropout occurred when the dropout rate was set as *q* = {0.3, 0.4, 0.5}.

(B) On the *m*

The *m* is an important parameter in machine learning that is set in a reasonable range; it can improve the memory utilization rate of the computer, improve its processing speed with the same amount of data, and reduce the training shock caused by the decline in memory. To observe the changes in the performance of the multilayer CNN model when the batch size was set to different values, we designed an experiment in this section. The parameter variable of this experiment was the batch size value; i.e., the other parameters were held constant: *q* = 0.5, *lr* = 1 × 10^−4^, and *ke* = 5. 

**Figure 3:**
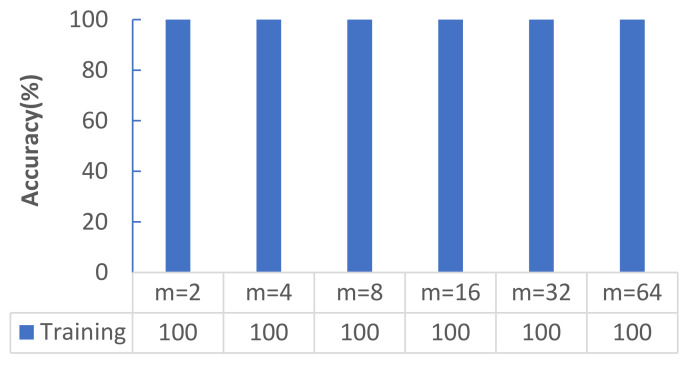
Training accuracies obtained under different *m* (%).

**Figure 4:**
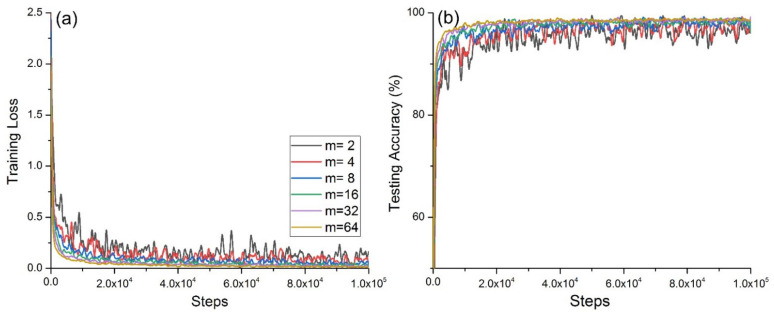
Model convergence under different *m*: (**a**) convergence of training cross-entropy loss; (**b**) convergence of test accuracy.

Figure 3 shows that with increasing *m*, the average accuracy rate did not change and reached 100%. Figure 4a shows the change in the cross-entropy loss with the same number of steps for the network model on the training set at different *m* values, and Figure 4b shows the accuracy of the network model on the test set with different *m*. Figure 4a shows that when *m* = 2, the convergence was slowest and exhibited strong oscillation; when *m* = 4, the convergence and oscillation were second only to those in the case when *m* = 2; when *m* = 64 and *m* = 32, the convergence was fast and the oscillation was small. Figure 4b shows that when *m* = 2, the convergence was slowest, and exhibited strong oscillation; when *m* = 4, its convergence and oscillation were second only to those in the case when *m* = 2; when *m* = 64 and *m* = 32, its convergence was fast, and the oscillation was small.

When *m* = {2,4,8,16}, the accuracy was high, but the training cross-entropy loss value and test accuracy oscillated greatly, and the convergence was poor. The reason for this was that the number of samples fed each time was too small, which led to overfitting during the model training process.

Table 2 shows that as *m* continued to increase, the running time for model also increased.

The above experimental results were in line with Equations (25) and (26). The *m* is one of the main hyperparameters that affects the learning performance of the deep model, and directly affects the parameter update of the cross-entropy loss function of the deep learning model.

Considering the convergence of the accuracy and cross-entropy loss during model training, the convergence of the accuracy during testing, and the running time incurred during the same steps, the experiment proved that when *m* = 32 was used, the performance of the deep neural network model reached its maximum.

(C) On the *lr*

The *lr* has a key impact on the performance of the model. Too large an *lr* causes the model to fail to converge, while a small rate causes the model to converge very slowly or fail to learn. To study the effects of different *lr* on the performance of a multilayer CNN, the parameter variables were *lr* = {1 × 10^−2^, 1 × 10^−3^, 1 × 10^−4^, 1 × 10^−5^, 1 × 10^−6^, 1 × 10^−7^}, while the other three parameters were held constant: *q* = 0.5, *m* = 32, and *ke* = 5. 

**Figure 5:**
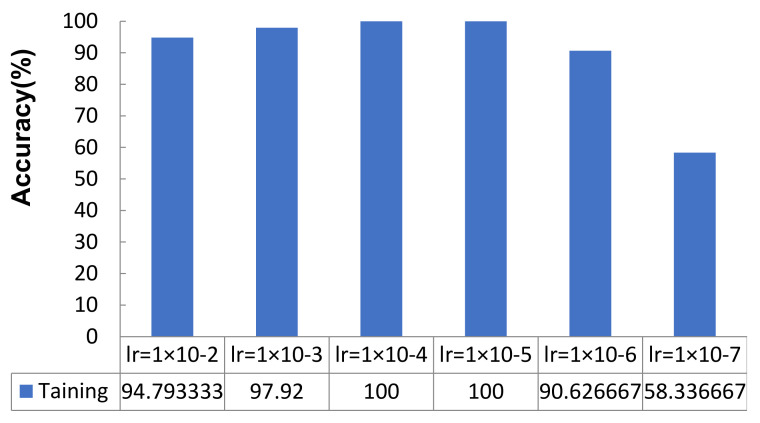
Training accuracies obtained under different *lr* (%).

It can be seen from Figure 5 that when the learning rate was in the range {1 × 10^−2^, 1 × 10^−3^}, the average accuracy rate increased with a decreasing learning rate; and when *lr* = 1 × 10^−4^ and *lr* = 1e-5, the average accuracy rate reached 100%. When *lr* < 1 × 10^−5^, the average accuracy dropped sharply, first to 90.6267%, and finally to 58.3367%.

**Figure 6:**
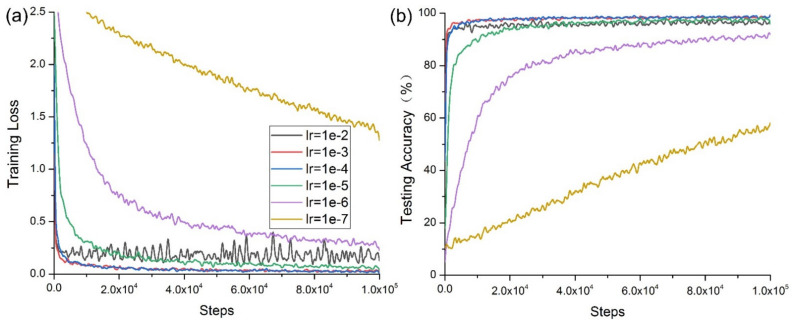
Model convergence with different *lr*: (**a**) convergence of training cross-entropy loss; (**b**) convergence of testing accuracy.

Figure 6 shows that when *lr* =1 × 10^−7^, both the training loss and testing accuracy failed to converge due to the low learning rate. When *lr* =1 × 10^−6^, the convergence rate of the model was slow, and was only faster than that obtained when *lr* =1 × 10^−7^. This experimental result is described in Equations (25) and (27); the learning rate is one of the main hyperparameters that affects the learning performance of the deep model and directly affects the parameter update of the cross-entropy loss function of the deep learning model. Figure 6b shows that the convergence rate of the deep CNN increased with an increasing learning rate. However, when *lr* = 1 × 10^−2^, its convergence worsened, and its oscillation increased. When going through the same steps, the time required by the model differed little, as shown in Table 3.

Experiments proved that the depth of the initial vector on CNN models had an impact on the performance, and when *lr* = 1 × 10^−3^ and *lr* =1 × 10^−4^, the depth of the neural network model attained the best performance condition.

(D) On the *ke*

The convolution process multiplies the elements in the convolution kernel and the corresponding pixels in the image successively, and sums them as the new pixel value after convolution. Then, the convolution kernel is shifted along the original image, and new pixel values are calculated until the whole image is covered. This process can produce many different effects according to different image convolution results.

In this section, experiments were designed to test the effects of different kernel sizes on the performance of the multilayer CNN. This set of experimental kernel sizes was used as a variable parameter, and its value was *ke* = {1,2,3,4,5,6,7}. The other three parameters were held constant: *q* = 0.5, *m* = 32, and *lr* =1 × 10^−4^.

**Figure 7:**
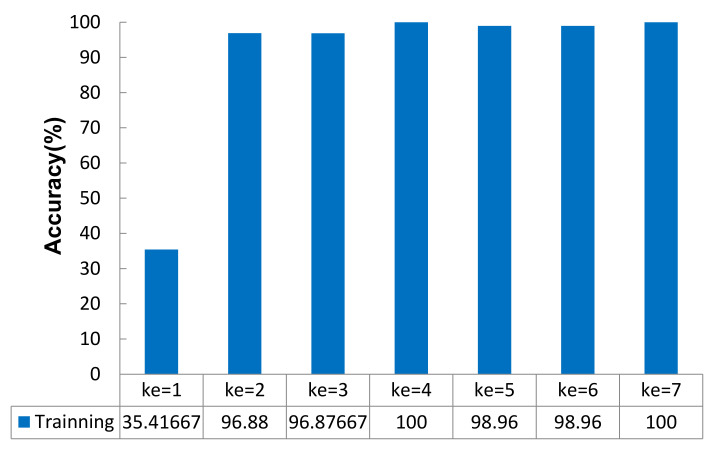
Training accuracies obtained under different *ke* (%).

When the other parameters of the network model remained unchanged, the accuracy value of the model changed in two stages with increasing kernel size: *ke* = {1,2,3,4} (stage 1) and *ke* = {5,6,7} (stage 2). The change trends in the two stages showed that the accuracy rate of the model increased with increasing kernel size, as shown in Figure 7.

**Figure 8:**
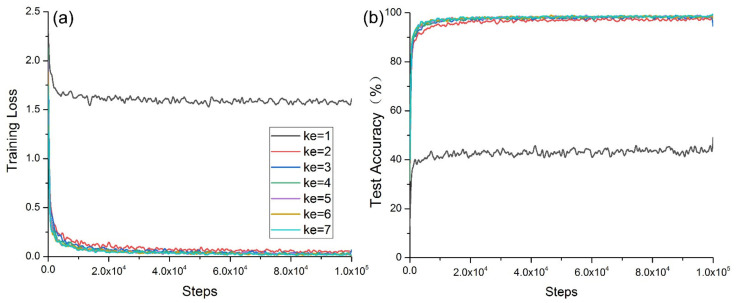
Model convergence with *ke*: (**a**) convergence of training cross-entropy loss; (**b**) convergence of test accuracy.

In Figure 8a, when *ke* = 1, the convergence was slowest and exhibited strong oscillation. When *ke* = 2, the oscillation was second only to those obtained when *ke* = 1. When *ke* > 3, its convergence was good, and there were no obvious changes in the kernel size. In Figure 8b, when *ke* = 1, the convergence was slowest and exhibited strong oscillation. When *ke* = 2, the convergence and oscillation were second only to those obtained when *ke* = 1. When *ke* > 3, the convergence was good, and there were no obvious changes in the *ke* value.

With an increasing *ke* value, the time consumed by the model for learning could be divided into two stages, as shown in Table 4. The time consumption at *ke* = 2 was 654 s lower than that at *ke* = 1. The time consumption at *ke* = 3 was 621 s lower than that at *ke* = 2. The time consumption at *ke* = 4 was 143 s higher than that at *ke* = 3. The time consumption at *ke* = 5 was 241 s higher than that at *ke* = 6. The time consumption at *ke* = 7 was 151 s higher than that at *ke* = 6.

According to the four indicators of model training accuracy, cross-entropy loss value convergence, test accuracy convergence, and the time spent during the same step, the experiments showed that *ke* had an impact on the performance of the deep CNN model. The optimal value range of the kernel size was *ke* = {4, 5}, and when *ke* = 4, the performance of the deep CNN model reached the best state.

### 5.5. Confirmatory Experiments and Analyses on Multiparameter Optimization

According to the experimental results obtained in Section 5.4 the *q*, *m*, *lr,* and *ke*, which enabled the model to achieve relatively optimal performances, were selected for combined experiments, and their network parameters are shown in Table 5.

**Figure 9:**
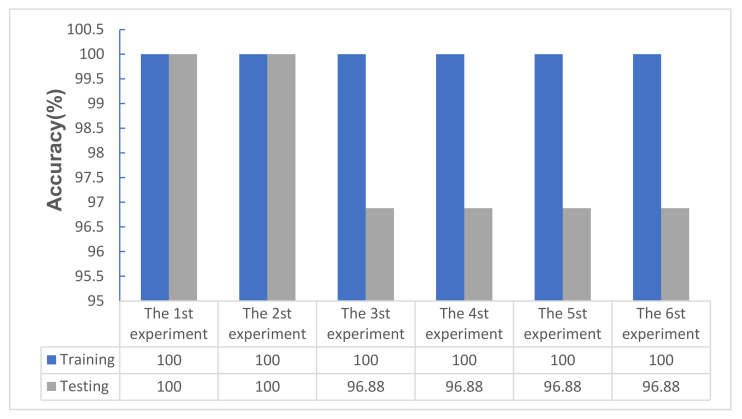
Training accuracies and test accuracies obtained for the confirmatory experiment (%).

**Figure 10:**
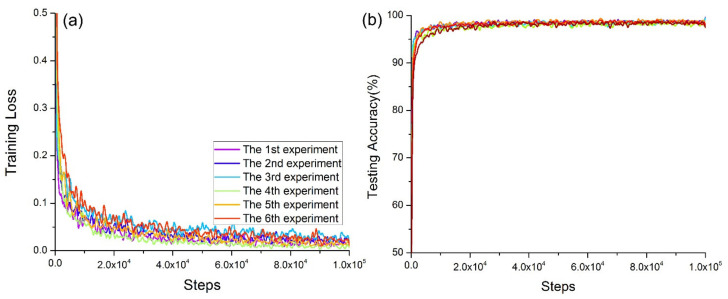
Model convergence for the confirmatory experiment: (**a**) convergence of training cross-entropy loss; (**b**) convergence of test accuracy.

Combining the relatively good dropout rate, batch size, learning rate, and convolution kernel size obtained in the experiments in Section 5.4, it was found that the parameter performance of the model was relatively stable at this time. The training accuracy and test accuracy were very similar (almost equal), as shown in Figure 9. However, in terms of convergence (as shown in Figure 10), the first group, the fourth group, and the second group had the best convergence rates, and the sixth group had the weakest convergence. The running time through the same steps appeared in the fifth group the least, followed by the first group and the fourth group. The differences between the time consumptions of the latter group and the time consumptions of the previous group were 29 s, 11 s, −34 s, −17 s, and 35 s (as shown in Table 6). With an improvement in computer performance, especially the performance of the GPU, if the time consumption difference yielded by the deep learning network model with the same number of steps was not large, the main performance indicators could be judged in terms of accuracy and convergence. Therefore, these verification experiments showed that the model had the best performance when the parameters of the model were set to the first group of parameters, namely, {*q*, *m*, *ke*, *lr*} = {0.3, 32, 4, 1 × 10^−3^}. The performance is best when the fourth group is used, that is, {*q*, *m*, *ke*, *lr*} = {0.3, 32, 4, 1 × 10^−4^}. The experimental results verified Equations (29) and (30). When the convolution kernel size and batch size were unchanged, the learning rate and discarding rate were the main parameters that affected the performance of the deep learning model, among which the learning rate had the greatest influence.

### 5.6. Repeatability Validation on Different Architectures

**Figure 11:**
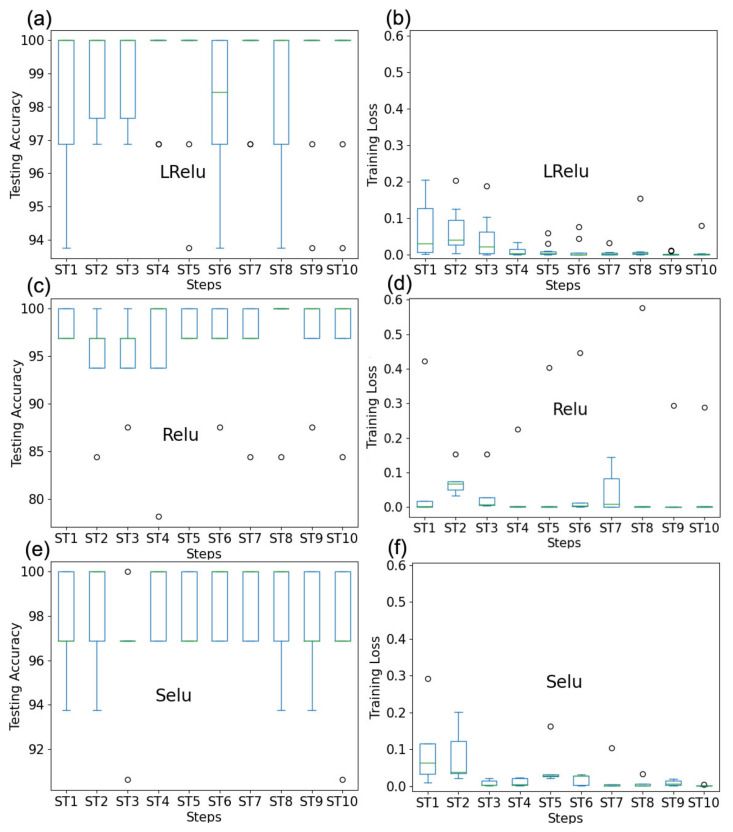
Statistical box plots of LRelu, Relu, and Selu models under different steps for 10 times experiment results: (**a**) testing accuracy of LRelu; (**b**) training loss of LRelu; (**c**) testing accuracy of Relu; (**d**) training loss of Relu; (**e**) testing accuracy of Selu; (**f**) training loss of Selu.

In order to avoid randomness of test results, we performed 10 repetitive cross-validation of the multiparameter experiment under the optimal hyperparameters ({*q*, *m*, *ke*, *lr*} = {0.3, 32, 4, 1 × 10^−4^}. It was guaranteed that they used the same training set for each case and for each variation of the hyperparameter studied. For each of these repetitions, the box diagram was statistically drawn for the results of every 10,000 steps, as shown in Figure 11. In particular, the {ST1 ST2, ST3, ST4, ST5 and ST6, ST7, ST8, ST9, ST10} corresponding steps were {10,000, 20,000, 30,000, 40,000, 50,000, 60,000, 70,000, 80,000, 90,000, 100,000}. Meanwhile, we selected two other activation functions, Relu and Selu, as different architectures to carry out experiments, which helped to validate and achieve stronger conclusions. It can be seen from Figure 11a,b that both the training loss function and the test accuracy rate could reach stability and convergence in the training process when the number of steps was higher than 900,000, which better verified the reliability of our experimental conclusions. To verify and draw convincing conclusions, we tested the above experimental values {*q*, *m*, *ke*, *lr*} = {0.3, 32, 4, 1 × 10^−4^} on the activation functions Relu and Selu, respectively, as shown in Figure 11c–f. It can be concluded that both the test accuracy rate and training loss function of the Relu and Selu model could show stability and convergence in the training process, which verified the reliability of our experimental conclusions. In other words, it indicated that the experimental results were plausible and were consistent with the conclusions of the mathematical derivation given in Section 4.

## 6. Conclusions

Although empirical and adaptive parameter-optimization models are often used to improve the performances of CNN models, empirical parameter models’ narrow limitations and adaptive parameter optimization models’ insufficient convergence rates or precondition constraints affect their applications and performances. This work attempted to provide analytical expressions for the mathematical relationships between the *q*, *m*, *lr,* and *ke* parameters and reveal the impacts of these four hyperparameters on the performance of a deep learning model in principle. It proposed a multiparameter optimization model for obtaining the optimal hyperparameters; the effectiveness of the proposed model was verified through various experiments based on the MNIST dataset, and the following conclusions could be drawn:

1. Firstly, from the perspective of single parameter, the interpretability of CNN was analyzed from two aspects of mathematical derivation and experimental verification. On this basis, an improved generalized optimization model considering multiple parameters was proposed to explain the CNN model and guide its parameter optimization, and it was verified by experiments. The mathematical equation that reflected the relationships between the *q*, *m*, *ke*, and *lr* hyperparameters could be concluded from the mathematical derivation and experimental verification: a. the learning performance was most sensitive to the *lr*, and cases in which the model did not converge, the convergence was too slow, or the model could not learn attributes were due to a learning rate that was too large or small; b. the *ke* had a certain impact on the performance of the model, and determined how efficiently the model learned features (the size of the output image) at each layer; and c. although the *m* and *q* were not as sensitive as the *lr*, they were also key parameters that affected model performance.

2. Twenty-eight sets of experiments were carried out by changing a single parameter each time, and we chose *q* = {0.1, 0.2, 0.3, 0.4, 0.5, 0.6, 0.7, 0.8, 0.9}, *m* = {2, 4, 8, 16, 32, 64}, and *lr* = {1 × 10^−2^, 1 × 10^−3^, 1 × 10^−4^, 1 × 10^−5^, 1 × 10^−6^, 1 × 10^−7^}. Experimental results showed that the best learning model performance could be obtained when the dropout rate, batch size, learning rate, and convolution kernel size were *q*= {0.3, 0.5}, *m* = {32, 64}, *lr* = {1 × 10^−4^, 1 × 10^−4^}, and *ke* = {4,5}, respectively. In particular, when the *q* = 0.4, *m* = 32, *ke* = 4, and *lr* = 1 × 10^−4^, the performance of the model was best.

3. The multi-hyperparameter mathematical correlation model proposed in this paper guided us to effectively perform the hyperparameter tuning process, which could also help us better understand deep learning models. Verification experiments showed that when the hyperparameters were set within reasonable ranges, the performance of the deep learning model was relatively stable, accurate, and convergent, so it could achieve relatively good results.

## Figures and Tables

**Table 1:** The running time required with the same number of steps for different *q*.

*q*	0.1	0.2	0.3	0.4	0.5	0.6	0.7	0.8	0.9
Running time (s)	10,094	10,118	10,270	10,023	10,089	10,240	10,192	10,041	10,060

**Table 2:** The running time required with the same number of steps at different *m*.

*m*	2	4	8	16	32	64
Running time (s)	1482	2101	2352	3474	5677	10,089

**Table 3:** The running time required with the same number of steps at different *lr*.

*lr*	1 × 10^−2^	1 × 10^−3^	1 × 10^−4^	1 × 10^−5^	1 × 10^−6^	1 × 10^−7^
Running time (s)	5596	5619	5677	5496	5554	5514

**Table 4:** The running time required for the same number of steps at different *ke*.

*ke*	1	2	3	4	5	6	7
Running time (s)	6719	6064	5443	5586	5677	5918	6069

**Table 5:** Network parameter design for the confirmatory experiment.

Experiment Number	*q*	*m*	*ke*	*lr*
1	0.3	32	4	1 × 10^−3^
2	0.4	32	4	1 × 10^−3^
3	0.5	32	4	1 × 10^−3^
4	0.3	32	4	1 × 10^−4^
5	0.4	32	4	1 × 10^−4^
6	0.5	32	4	1 × 10^−4^

**Table 6:** The running time required for the same number of steps.

Experiment Number	1st Experiment	2nd Experiment	3rd Experiment	4th Experiment	5th Experiment	6th Experiment
Running time (s)	5298	5337	5358	5314	5297	5332

## Data Availability

The MNIST datasets are available at http://yann.lecun.com/exdb/mnist/ (accessed on 22 February 2021).

## Abbreviations

**Symbol**	**Description**
*q*	Dropout rate
*lr*	Batch size
*m*	Learning rate
*ke*	Convolution kernel size (kernel height = kernel width)
*K*	Number of sample categories
*X*	Sample dataset
$z^{\left(l\right)}$	Vector input to the *l* layer
$y^{\left(l\right)}$	Output vector from the *l* layer
f(z)	Activation function
$x_{i}^{\left(l\right)}$	Input of the *l* layer
$w_{i}^{\left(l\right)}$	Weight of the *l* layer of $x_{i}^{\left(l\right)}$
$b_{i}^{\left(l\right)}$	Bias of the *l* layer of $x_{i}^{\left(l\right)}$
${\vec{x}}_{i,k}^{\left(l\right)}$	Input of the sample of the K category in the *l* layer
$w_{k,i}^{\left(l\right)}$	Weight of the *l* layer of class k
$b_{k,i}^{\left(l\right)}$	Bias of the *l* layer of class k
I	Input image size
S	Stride size
P	Padding size
O	Output image size
$W_{c}$	Number of weights the convolutional layer
$B_{c}$	Number of biases of the convolutional layer
$P_{c}$	Number of all parameters of the convolutional layer
N	Set number of cores
CH	Number of input image channels
$W_{cf}$	Number of biases of the fully connected layer connected to the convolutional layer
$B_{cf}$	Number of weights of the fully connected layer connected to the convolutional layer
$o^{\prime }$	Output image size of the previous convolution layer
$P_{cf}$	Total number of parameters in the current fully connected layer
*F*	Number of neurons in the current fully connected layer
*F* *_−1_*	Number of neurons in the previous fully connected layer
